# Androgen Deprivation Therapy in Patients With Prostate Cancer Increases Serum Levels of Thromboxane A_2_: Cardiovascular Implications

**DOI:** 10.3389/fcvm.2021.653126

**Published:** 2021-04-13

**Authors:** Mario Álvarez-Maestro, Aritz Eguibar, Patricia Chanca, Mercedes Klett-Mingo, Juan Gómez Rivas, Antonio Buño-Soto, Fermín R. de Bethencourt, Mercedes Ferrer

**Affiliations:** ^1^Servicio de Urología, Hospital Universitario La Paz, Madrid, Spain; ^2^Grupo de Investigación en Urología, IdiPAZ, Madrid, Spain; ^3^Servicio de Análisis Clínicos, Hospital Universitario La Paz, Madrid, Spain; ^4^Departamento de Fisiología, Facultad de Medicina, UAM, Madrid, Spain; ^5^Departamento de Urología, Hospital Clínico San Carlos, Madrid, Spain; ^6^Grupo de Investigación en Neonatología, IdiPAZ, Madrid, Spain

**Keywords:** prostate cancer, androgen deprivation therapy (ADT), thromboxane A_2_ (TXA_2_), vascular function, endothelium

## Abstract

**Introduction:** Androgens have been described as important players in the regulation of vascular function/structure through their action on the release and effect of vasoactive factors, such as prostanoids. Patients with prostate cancer (PCa) under androgen deprivation therapies (ADTs) present increased risk of cardiovascular mortality. Since thromboxane A_2_ (TXA_2_) is one of the most studied prostanoids and its involvement in different cardiovascular diseases has been described, the aim of this study was to investigate: (i) the effect of ADT on the serum levels of TXA_2_ in PCa patients and its possible link to the redox status and (ii) the effect of the non-hydrolyzable TXA_2_ analog U-46619 on the function of the aorta of male rats.

**Methods:** The levels of TXA_2_ and total antioxidant status in 50 healthy subjects, 54 PCa patients, and 57 PCa under ADT were evaluated. These determinations were accompanied by levels of testosterone and C-reactive protein as an inflammation marker. In aortic segments from male rats, the U46619-induced effects on: (i) the vasomotor responses to acetylcholine (ACh), to the NO donor sodium nitroprusside (SNP), to the carbon monoxide-releasing molecule-3 (CORM-3), and to noradrenaline (NA) and (ii) the expression of cyclooxygenase-2 (COX-2), heme oxygenase-1 (HO-1), and phosphorylated ERK1/2 were analyzed.

**Results:** The serum level of TXA_2_ in patients with PCa was increased with respect to healthy subjects, which was further increased by ADT. There was no modification in the total antioxidant status among the three experimental groups. In aortic segments from male rats, the TXA_2_ analog decreased the endothelium-dependent relaxation and the sensitivity of smooth muscle cells to NO, while it increased the vasoconstriction induced by NA; the expression of COX-2, HO-1, and pERK1/2 was also increased.

**Conclusions:** ADT increased, along with other inflammatory/oxidative markers, the serum levels of TXA_2_. The fact that TXA_2_ negatively impacts the vascular function of the aorta of healthy male rats suggests that inhibition of TXA_2_-mediated events could be considered a potential strategy to protect the cardiovascular system.

## Introduction

Prostate cancer (PCa) is one of the most important leading causes of cancer deaths in men worldwide (1, 2). Androgen deprivation therapy (ADT) is the most widely used treatment for advanced PCa, which aims to reduce the levels and function of androgens to prevent PCa growth and spread (3). However, ADT is associated with several adverse side effects including osteoporosis, fatigue, depressive symptoms, sexual dysfunction, and metabolic modifications (4). The metabolic changes linked to ADT include altered lipid profile, insulin resistance, increase in adipose tissue, and adipokines (5), which favors a pro-inflammatory and pro-oxidant environment, giving rise to the so-called metabolic syndrome (6, 7), which is a cluster of risk factors for cardiovascular diseases. Indeed, several observational trials have reported an increased risk of cardiovascular diseases in men with PCa on ADT (8–10). Likewise, an association between lower plasma levels of testosterone and hypertension has been reported (11–15).

These clinical observations have been reinforced with experiments performed in different animal models demonstrating that decreased levels of testosterone alter vascular function and structure by modulating lipid profile (16); the release of endothelial factors such as nitric oxide (NO), prostanoids, and reactive oxygen species (ROS) (17–19); and different cell signaling pathways (20, 21). Among prostanoids, thromboxane A_2_ (TXA_2_) has been implicated in the development of cardiovascular diseases such as hypertension (22, 23) and thromboembolic events (24, 25). Since TXA_2_ is able to modulate the production of NO (26) and ROS (27, 28) as well as to activate vascular remodeling (29), mechanisms that, if maintained for a long time, can lead to different vascular pathologies, the first objective was to analyze the effect of ADT in PCa patients on the serum levels of TXA_2_ and its possible link to the redox status. The second objective was to explore the possible detrimental action of TXA_2_ on vascular function of aortic segments of male rats by analyzing the effect of the non-hydrolyzable TXA_2_ analog U-46619 on the vasodilator and vasoconstrictor responses.

## Materials and Methods

### Participants and Study Design

This is a prospective cohort study whose participants were patients in the Department of Urology of the La Paz University Hospital. All participants gave written informed consent. The study protocol was approved by the local Clinical Research Ethics Committee (Ref. HULP: PI-1204).

Participants were divided into the following three groups: healthy group (56 participants without PCa), PCa group (55 patients with localized PCa), and PCa+ADT (59 advanced PCa patients treated with ADT at least for 6 months and with testosterone concentration to castration levels during measurement defined by a serum testosterone concentration below 50 ng/dl). In the two groups of patients with PCa, the cancer was confirmed by standard prostate biopsy procedure. Systolic/diastolic blood pressure and heart rate were measured in all participants. Participants under medication for the treatment of hypertension, diabetes, or dyslipidemia were excluded.

### Thromboxane A_2_, Total Antioxidant Capacity, and Other Biomarkers

Fasting blood was collected coinciding with a health-care blood extraction. Once the serum samples were obtained, they were stored at −80°C until used. The content of TXA_2_ was analyzed by measuring its stable metabolite TXB_2_ by enzyme immunoassay (Fine Test). The total antioxidant capacity in serum samples was analyzed by using the hydrophilic oxygen radical scavenging capacity (ORAC) assay (Randox Laboratories). Levels of prostate-specific antigen (PSA), follicle-stimulating hormone (FSH), luteinizing hormone (LH), testosterone, and estradiol were measured by chemiluminescence immunoassay in an Advia Centaur analyzer (Siemens Healthineers). The assays were carried out according to the manufacturer's protocols. Glucose and uric acid were measured by enzymatic-spectrophotometric methods in an Advia 2400 analyzer (Siemens Healthineers) and C-reactive protein (CRP) by immunoturbidimetric method in an Advia 2400 analyzer (Siemens Healthineers).

### Animals and Vascular Tissue Preparation

Male Sprague–Dawley rats, 5 months old, were provided by the Animal Facility of the Universidad Autónoma de Madrid (UAM) (Registration number EX-021U). Systolic blood pressure was indirectly measured in awake animals by the tail-cuff method (Letica, Digital Pressure Meter, LE5000, Barcelona, Spain), and the animals were weighted before sacrifice. Rats were sacrificed by CO_2_ inhalation and subsequent decapitation, and the thoracic aorta was carefully dissected out, cleaned of connective tissue, and placed in Krebs–Henseleit solution (KHS) at 4°C. The composition of KHS is as follows (mM): NaCl 115, CaCl_2_ 2.5, KCl 4.6, KH_2_PO_4_ 1.2, MgSO_4_·7H_2_O 1.2, NaHCO_3_ 25, glucose 11.1, and Na EDTA 0.03. All animal protocols were approved by the Research Ethics Committee of UAM according to directives 609/86 CEE and R.D. 233/88 of the Ministerio de Agricultura, Pesca y Alimentación of Spain (PROEX 202/16). The experiments were conducted in accordance with the published Guiding Principles in the Care and Use of Animals approved by the European Union directives 63/2010 UE and Spanish regulation RD53/2013.

### Vascular Reactivity

The method used for isometric tension recording has been described in full elsewhere (30). Briefly, aortic segments were suspended in an organ bath containing 5 ml of KHS at 37°C, continuously bubbled with 95% O_2_-5% CO_2_ mixtures (pH 7.4). Two parallel stainless steel pins were introduced through the lumen of the vascular segment: one fixed to the bath wall and the other connected to a force transducer (Grass FTO3C; Grass Instruments Co., Quincy, MA, USA); this in turn was connected to a model 7D Grass polygraph. The aortic segments were subjected to a tension of 1 g, which was re-adjusted every 15 min during a 90-min equilibration period before drug administration. After this, the vessels were exposed to 75 mM of KCl to check the functional integrity. After a washout period, the viability of vascular endothelium was tested by the ability of 10 μM of ACh to relax precontracted segments with 0.1 μM of noradrenaline (NA). Vessels were then washed with KHS to recover the basal tension. To investigate the effect of the non-hydrolyzable TXA_2_ analog U-46619 on the vasomotor responses, separate aortic segments of SD rats were incubated with 1 nM of U-46619 for 1 h before performing cumulative concentration–response curves to ACh (0.1 nM−10 μM), to the NO donor sodium nitroprusside (SNP, 0.1 nM−10 μM), to the carbon monoxide-releasing molecule-3 (CORM-3, 1 μM−0.1 mM), and to NA (0.1 nM−10 μM). The concentration of the TXA_2_ analog (1 nM) was chosen because it was within the range of the intracrine concentration reached in the vascular wall that we had previously reported (26). Thus, we described that the release of TXA_2_ in the aorta from control and orchidectomized rats varied from 100 to 400 pg/ml/mg tissue.

### Western Blotting Analysis

Arterial segments from the two experimental conditions (control and exposed to 1 nM of U-46619) were homogenized and processed to quantify protein concentration at 4°C in radioimmunoprecipitation assay (RIPA) buffer containing phosphatase inhibitors and a cocktail of protease inhibitors. Proteins (20 μg) were separated by sodium dodecyl sulfate–polyacrylamide gel electrophoresis (SDS-PAGE) gels and transferred to polyvinylidene difluoride (PVDF) membranes (Bio Rad Immun-Blot® overnight at 4°C, 230 mA, using a Bio-Rad Mini Protean III system; Bio-Rad Laboratories, Hercules, CA, USA). Membranes were blocked with 5% (w/v) fat-free powdered milk or 5% (w/v) bovine serum albumin following the instructions of the antibody manufacturers and incubated overnight with mouse monoclonal antibody for anti-phospho-ERK1/2–Thr202/Tyr204 (1:2,000 dilution, Cell Signaling Technology) or with rabbit polyclonal antibody for cyclooxygenase-2 (COX-2) (1:200 dilution, Cayman Chemical) or heme oxygenase (HO-1) (1:2,000 dilution, Stressgen Bioreagents). After being washed, the membrane was incubated with the corresponding anti-immunoglobulin G conjugated to horseradish peroxidase (Amersham International Plc). The membrane was thoroughly washed, and the immunocomplexes were detected using an enhanced horseradish peroxidase/luminol chemiluminescence system (ECL Plus, Amersham International Plc, Little Chalfont, UK) and subjected to autoradiography (Hyperfilm ECL, Amersham International Plc). Signals on the immunoblot were quantified using a computer program (NIH Image V1.56). The same membrane was used to determine GAPDH expression, and the content of the latter was used to correct COX-2 and HO-1 expression in each sample, by means of a monoclonal antibody anti GAPDH (1:5,000 dilution, Sigma). The total ERK1/2 was used as loading control to correct the phosphorylation level of ERK1/2.

### Drugs and Chemicals

Drugs used were as follows: ACh chloride, potassium chloride, SNP, CORM, and l-NA hydrochloride (Sigma-Aldrich). Stock solutions (10 mM) of drugs were prepared in distilled water, except for NA, which was dissolved in NaCl (0.9%)–ascorbic acid (0.01% w/v) solution. These solutions were kept at −20°C, and appropriate dilutions were made in KHS on the day of the experiment.

### Data Analysis

The results carried out on human participants are expressed as the mean with the standard deviation or by the mean value and the corresponding 25 and 75th percentiles. For comparison among the three groups, in the case of quantitative variables, with normal distribution, the one-way ANOVA test was used. Then, a *post-hoc* contrast was carried out to analyze the groups with different values. If the distribution of the variable is not normal, non-parametric tests as Kruskal–Wallis H Mann–Whitney *U*-tests were used. The normal distribution of quantitative variables was checked using the Shapiro–Wilk test. To analyze the associations between the levels of two variables, the Spearman coefficient (ρ) was used. A two-tailed *p* < 0.05 was considered statistically significant. The statistical analysis was performed using the statistical packages SPSS version 26.0 and Stata version 16.0.

The results of animal experiments are given as mean ± standard error of the mean (SEM). The relaxation induced by ACh, SNP, or CORM-3 was expressed as a percentage of initial contraction elicited by NA. The contraction induced by NA was expressed as percentage of the contraction induced by KCl (75 mM). Statistical analysis was performed by comparing the curves obtained in aortae of SD rats after U-46619 incubation with that obtained in the control condition by means of two-way analysis of variance (ANOVA). For protein expression, statistical analysis was done using Student's *t*-test for unpaired experiments. A *p* < 0.05 was considered significant.

## Results

### Effect of Androgen Deprivation Therapy in Prostate Cancer Patients on Serum Levels of Thromboxane A_2_, Total Antioxidant Activity, and Other Biomarkers

The characteristics of the three groups of subjects are summarized in Table 1. The patients of the ADT group were older than those of PCa or healthy groups (*p* < 0.001); patients belonging to PCa+ADT group showed the greatest values for systolic blood pressure (*p* < 0.02) and heart rate (*p* < 0.05) as compared with PCa or healthy groups.

**Table 1:** Characteristics of the study population.

	**Healthy**	**PCa**	**PCa^+^ADT**	***p***
	**(*n* = 56)**	**(*n* = 55)**	**(*n* = 59)**	
Age (years)	68.77 (10.47)	69.69 (9.22)	77.90 (8.08)^*^	<0.001
DBP (mmHg)	72.51 (8.81)	71.29 (9.66)	74.12 (6.34)	NS
SBP (mmHg)	126.15 (11.03)	127.09 (10.14)	131.02 (7.19)^*^	0.01
HR (bpm)	73.25 (6.94)	72.22 (7.83)	75.78 (6.65)^*^	0.03
PSA (ng/ml)	1.18 (0.52)	1.13 (0.38)	3.23 (2.49)^*^^+^	0.009
FSH (mUI/ml)	14.00 (12.66)	10.67 (6.48)	5.96 (7.66) ^*^^+^	<0.001
LH (mUI/ml)	7.99 (12.17)	5.75 (3.57)	1.04 (3.75)^*^^+^	<0.001
Test (pg/ml)	6.22 (13.50)	14.91 (6.74)	1.89 (3.82)^*^^+^	<0.001
Estr (pg/ml)	11.62 (29.50)	32.20 (11.65)	17.93 (9.54)^*^^+^	<0.001
Glucose (mg/dl)	109.38 (25.71)	111.85 (30.06)	112.58 (32.44)	NS
Uric acid (mg/dl)	6.25 (1.96)	5.60 (1.22)	5.00 (1.36)^*^	<0.001
CRP (mg/dl)	3.08 (3.88)	3.12 (4.45)	8.29 (26.48)	NS
TXA_2_ (pg/ml)	13.02 (8.52)	17.81 (11.06)^*^	26.96 (43.78) ^*^	<0.001

*Values are expressed as mean with standard deviation in parentheses*.

* *Statistical significance compared with healthy group*.

+ *Statistical significance compared with PCa group*.

*DBP, diastolic blood pressure; SBP systolic blood pressure; HR, heart rate; PSA, prostatic specific antigen; FSH, follicle-stimulating hormone; LH, luteinizing hormone; Test, testosterone; Estr, estradiol; CRP, C-reactive protein; TXA_2_, thromboxane A_2_*.

The levels of PSA were similar between healthy and PCa groups, while they were increased in the PCa+ADT group (*p* < 0.01). The levels of FSH, LH, estradiol, and testosterone were reduced in the PCa+ADT group compared with PCa (*p* < 0.001) and healthy (*p* < 0.001) groups. The glucose concentration was similar in the three groups of the study. The concentration of uric acid was decreased in the PCa+ADT group with respect to the healthy group (*p* < 0.001). There were no statistical differences for CRP among groups.

**Figure 1:**
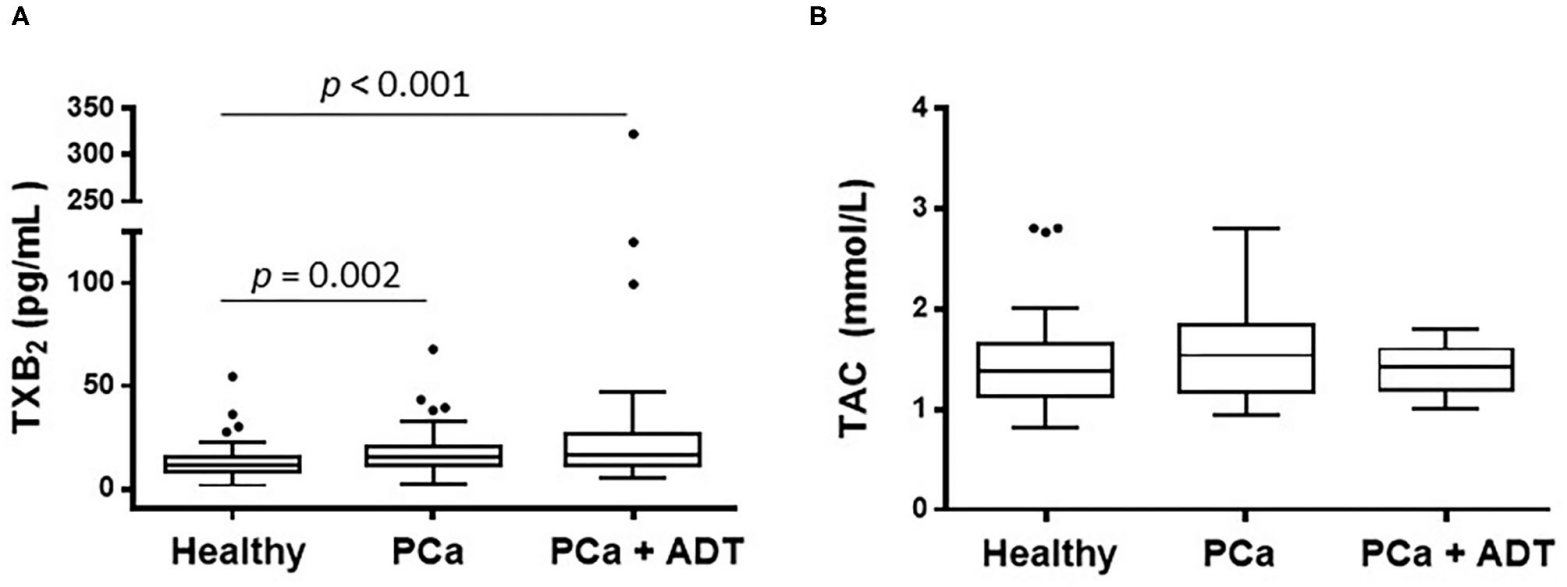
Effect of prostate cancer (PCa) and androgen deprivation therapy (ADT) in PCa patients on the serum concentration of thromboxane A_2_ (TXA_2_) **(A)** and on the total antioxidant capacity (TAC) **(B)**. Results are shown as the median (solid line) with the top and bottom of box representing quartiles. The statistical significance, by means of Mann–Whitney U test, is indicated in the corresponding graphs.

The patients belonging to PCa group presented higher serum levels of TXA_2_ than those of the healthy group; those levels were further higher in PCa patients under ADT (Figure 1A and Table 1); the total antioxidant capacity was similar in the three groups of the study (Figure 1B).

The concentration of TXA_2_ was inversely correlated with the concentration of testosterone or estradiol and directly correlated with glucose concentration (Table 2). In addition, a direct correlation between total antioxidant capacity and uric acid was observed (ρ: 0.185; *p* = 0.01).

**Table 2:** Correlation between TXA_2_ and testosterone, estradiol, or glucose.

**TXA_**2**_**	**ρ**	***p***
Testosterone	−0.20	0.007
Estradiol	−0.25	0.001
Glucose	0.22	0.003

*ρ indicates Spearman coefficient*.

### Effect of the Non-hydrolyzable Thromboxane A_2_ Analog on Vascular Function of Rat Aorta

**Figure 2:**
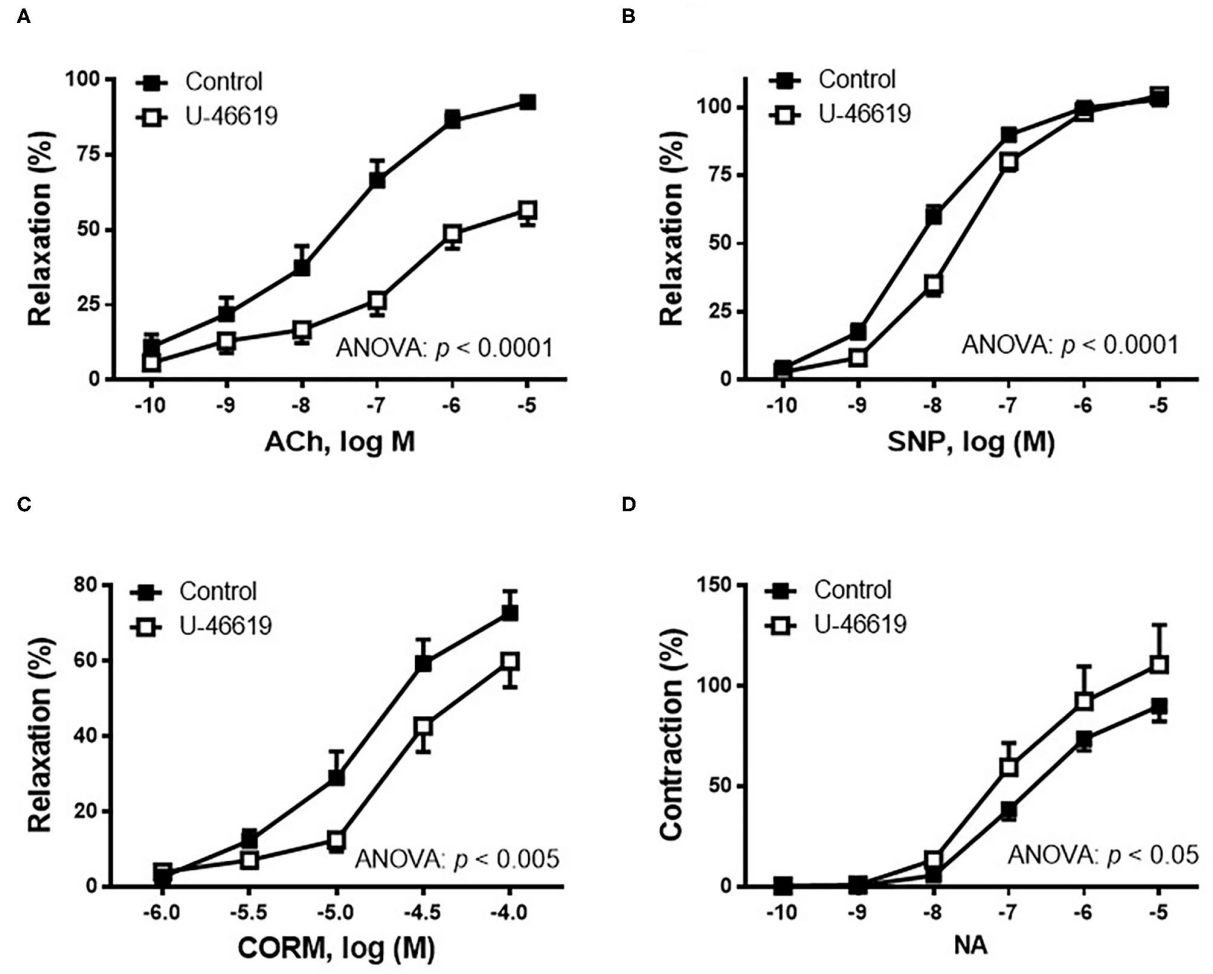
Effect of the non-hydrolyzable mimetic of thromboxane A_2_ (TXA_2_), U-46619 (1 nM), on the concentration–response curves to **(A)** acetylcholine (ACh), **(B)** sodium nitroprusside (SNP), **(C)** carbon monoxide-releasing molecule (CORM), and **(D)** noradrenaline (NA) in aortic segments of male rats. Results (mean ± SEM) are represented for the vasodilatory responses as percentage of the inhibition of the contraction elicited by 0.1 μM of noradrenaline; for the NA-induced vasoconstriction, the response was represented as percentage of the previous contraction induced by KCl (75 mM). Number of animals: 4–5. The statistical significance is indicated in the corresponding graphs.

The effect of TXA_2_ mimetic on the endothelium-dependent vasodilator response was analyzed. Therefore, in NA-precontracted arterial segments, the vasodilator response induced by ACh (0.1 nM−10 μM) was decreased after U-46619 incubation (Figure 2A). Since NO is one of the most important factors released after endothelial stimulation, the possible action of the TXA_2_ mimetic on the sensitivity of smooth muscle cells to NO was also investigated by analyzing the vasodilator response induced by the NO donor, SNP. The results showed that in NA-precontracted arteries, the vasodilator response induced by SNP (0.1 nM−10 μM) was decreased after U-46619 incubation (Figure 2B).

Since carbon monoxide (CO) has been described to elicit cytoprotective actions in responses to cellular stress, the effect of U-46619 on the CORM-induced vasodilator response was also analyzed. The results showed that the vasodilator response to CORM (1–100 μM) was decreased after incubation with 1 nM of U-46619 (Figure 2C).

The contractile response elicited by 75 mM of KCl was not modified after incubation with then non-hydrolyzable mimetic of TXA_2_, U-46619 (control: 1,988 ± 184.5 mg; 1 nM of U-46619: 2,143 ± 139.0 mg; *p* > 0.05). The vasoconstrictor response induced by NA (0.1 nM−10 μM) was increased after incubation of vessels with U-46619 (Figure 2D).

**Figure 3:**
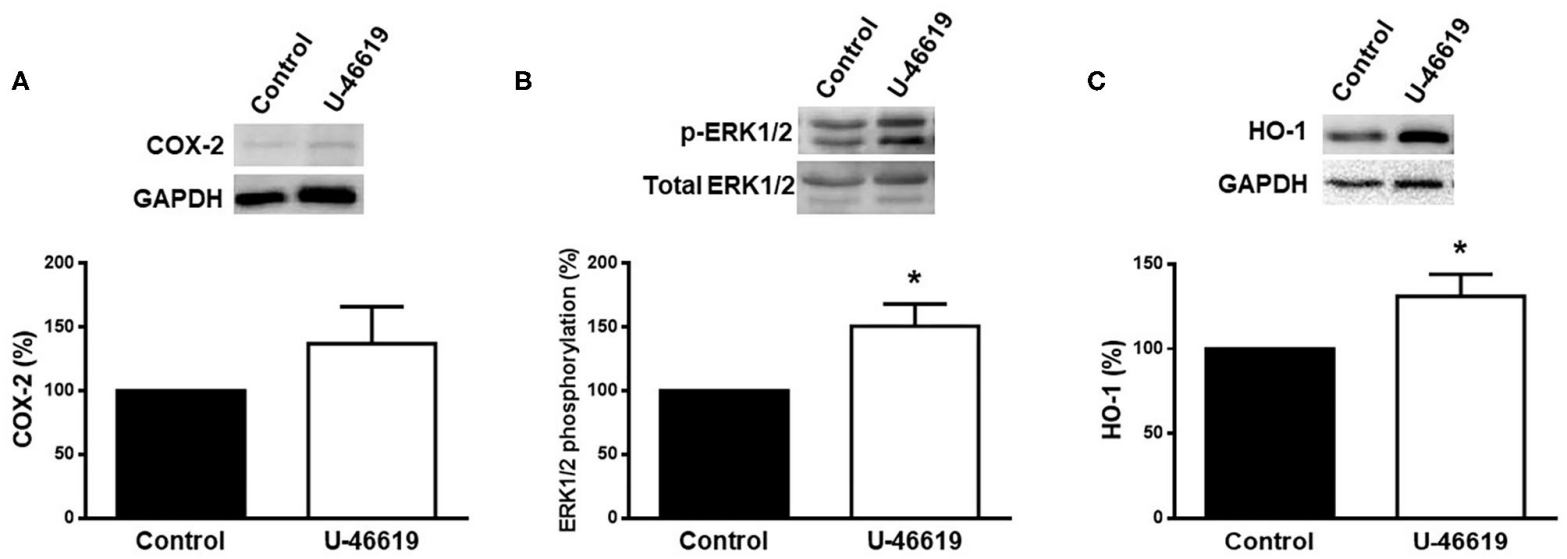
Representative western blot and densitometric analysis for the expression of COX-2, p-ERK1/2, and HO-1 protein in aortic segments of male rat in the absence (control) or presence of U-46619 (1 nM). Results (means ± SEM) are expressed as the relative percentage of the ratio between the signal for COX-2 or HO-1 protein and the signal for GAPDH; the signal for p-ERK1/2 was corrected to the corresponding total ERK1/2 signal. Number of animals: 3. *p < 0.05 compared with control condition.

The effect of U-46619 on the expression of COX-2, p-ERK1/2, and HO-1 was analyzed in homogenates of rat aorta by using western blot analysis. The results show that the expression of COX-2 was weakly increased (*p* > 0.05), while that of pERK1/2 and HO-1 was significantly increased after U-46619 incubation (Figure 3).

## Discussion

The present work describes for the first time that patients with PCa show increased serum levels of TXA_2_ and that ADT further increases those levels, which may account for the development of vascular dysfunction. In addition, the detrimental effect of the non-hydrolyzable TXA_2_ analog, U-46619, in an *in vitro* model is also demonstrated.

It is well-known that TXA_2_, through its T prostanoid receptor (TP), has been implicated in the progression of different cancers including PCa (31–33), which suggests the involvement of inflammatory pathways in PCa (34). Although higher circulating TXA_2_ levels have been associated with colorectal cancer progression (35), to our knowledge, there is no information on circulating TXA_2_ levels in PCa patients. The current study showed that serum TXA_2_ levels are increased in the PCa group with respect to healthy subjects. This finding is in agreement with that describing the involvement of prostanoids and chronic inflammation in carcinogenesis (36) and with the fact that aspirin intake reduced the PCa incidence (37). According to the literature (3, 38), administration of ADT achieves reduced levels of FSH, LH, and testosterone in PCa patients to avoid tumor growth and spread. Although ADT has been shown to improve survival, deleterious effects of ADT on cardiometabolic risk (4) and type 2 diabetes mellitus (39) have been reported. In addition, patients with these pathologies present chronic inflammation and increased synthesis of TXA_2_ (40). Despite that TXA_2_ has been implicated in the pathogenesis of a variety of cardiovascular diseases (41, 42), the impact of ADT on this prostanoid has not been still investigated. The results described in the current study showed that in PCa patients on ADT, the levels of TXA_2_ were strongly increased, showing an inverse correlation between the levels of testosterone and TXA_2_. This finding is consistent with data from other studies, which reported that administration of androgens to patients with coronary heart disease decreased the TXA_2_ level (43), while the loss of gonadal function of male rats increased the levels of TXA_2_ (18, 19, 26). Our results also showed that serum TXA_2_ concentration was directly correlated with glucose concentration, while it was not correlated with CRP, a chronic inflammation marker. It is important to mention that CaP patients on ADT showed higher values of CRP than did healthy and PCa groups, although the difference was not statistically significant. In this sense, long-term testosterone therapy of hypogonadal men decreased the level of CRP (15).

Since TXA_2_ is able to increase the synthesis of ROS (22, 27), which, in turn, decreases the bioavailability of NO and therefore may account for detrimental actions on cardiovascular function (16, 44), total antioxidant capacity was analyzed in the three experimental groups. Contrary to what was expected, the results showed that there were no statistical differences among the groups. A possible explanation is that the age of the participants is high, even in the healthy group; therefore, the antioxidant capacity could already be diminished according to previously reported data in aging (45). It is interesting to mention that total antioxidant capacity was directly correlated with the serum uric acid concentration, which was decreased in the PCa patients on ADT with respect to the healthy subjects. Still within the normal range, the increased levels of uric acid observed in the healthy group could be considered as a compensatory mechanism to counteract the oxidative stress related to aging because of its antioxidant property, as reported in a variety of pathophysiological conditions (46, 47). Despite the limitation of this study, which lacks healthy young participants and that precisely the PCa patients on ADT are significantly older than the other groups, undoubtedly, ADT increased thromboxane levels. Therefore, stratified studies—according to age and to ADT duration—analyzing in detail the potential association between TXA_2_ levels and different cardiovascular events should be of great interest to be performed in the future.

The current study revealed that TXA_2_ was able to induce dysfunction in rat aorta. The *in vitro* model consisted of incubating aortic rings of male rats with the non-hydrolyzable TXA_2_ analog, U-46619, according to previous investigations (27, 44). The results showed that 1 nM of U-46619 for 1 h decreased the endothelium-dependent response elicited by ACh. It is well-known that ACh induces the release of vasodilators factors including NO and hyperpolarizing factors (16, 48). This result could be compatible with a decreased release and/or bioavailability of NO induced by TXA_2_. In this sense, endogenous TXA_2_ was reported to negatively regulate the NO release in mesenteric artery of male rats (26). TXA_2_ is also able to decrease NO bioavailability through increasing the synthesis of ROS (28, 49). In addition, the possible modulation of TXA_2_ on the sensitivity of smooth muscle cells to NO was also analyzed. The results showed that the SNP-induced response was decreased by incubation with 1 nM of U-46619, which appears to agree with studies describing that endogenous TXA_2_ negatively modulates the vasodilator effect of NO (26). The underlying mechanism could be due to the inhibition of guanylate cyclase by U-46619 as reported in radial arteries (50).

Since NO and cGMP can hyperpolarize cell membranes by activating potassium channels (51, 52), possible modifications in the function of these channels should not be ruled out. Another important gas mediator activating potassium channels is CO, which is produced by heme oxygenases during the degradation of the heme group. The inducible isoform of HO-1 is one of the earliest expressed proteins in response to inflammation and oxidative stress, and its involvement in cardiovascular protection has been described (53, 54). In this regard, our results showed increased HO-1 expression in incubated arteries with U-46619, supporting the pro-inflammatory environment induced by the TXA_2_ analog. This response could be due, at least in part, to the action of products derived from COX-2—although COX-2 was slightly increased—and from the extracellular signal-regulated kinase (ERK1/2). Thus, reduction of TXA_2_ synthesis has been reported to prevent of ERK1/2 phosphorylation and prostaglandin E_2_ effect in human monocytes (55). Once the up-regulation of HO-1 was observed in arteries incubated with U-46619, the next step was to analyze the possible influence on the vasodilatory effect of CO. The fact that the CORM-induced relaxation was decreased by U-46619 incubation suggests a reduction of the potassium channels functionality. In this regard, the blockage of potassium channels by TP receptors activation in pulmonary arteries was described (27).

It is well-known that TP receptor activation results in the stimulation of intracellular pathways including phospholipase C with the subsequent production of 1,4,5-triphosphate and diacylglycerol and, therefore, increase in calcium release from sarcoplasmic reticulum and protein kinase activation (56). Activation of the above mentioned cell-signaling pathways potentiate the vasomotor response to several vasoconstrictor agents (57). This observation could explain the increased NA-induced response observed in the current study, which agrees with previous investigations describing that U-46619 facilitates sympathetic neurotransmission and potentiates constrictor effects of NA in human saphenous veins (58).

It is remarkable to note that most of published studies use higher concentrations than 1 nM of U-46619 used in the current investigation, which supports the relevance of the functional results observed in rat aorta. Although 1 nM of TXA_2_ analog is around 10-fold higher than the concentration observed in the serum of PCa patients under ADT, it is similar to the intracrine concentration reached in the vascular wall (26). The results obtained from the *in vitro* model showed a TXA_2_-induced detrimental effect on rat aorta, in which endothelium-dependent and endothelium-independent vasodilation was compromised. These results suggest a deleterious effect of TXA_2_ on vascular function as consequence of ADT, which could support the slight increase in blood pressure observed in the PCa+ADT group. Although our study did not measure the incidence of *de novo* cardiovascular events, ADT has shown increased cardiovascular risk (4), increase in diabetes mellitus (39), myocardial infarction, sudden cardiac death (59), and thromboembolic events (60, 61). Conducting stratified studies by age range of patients and by ADT duration would improve the analysis about the ADT effects on different biomarkers and cardiovascular events.

## Conclusion

Overall, the current study showed that PCa patients on ADT increased the serum levels of TXA_2_, which could exert detrimental effects on the cardiovascular system. Our *in vitro* results showed that TXA_2_ negatively impacts the function of the aorta of healthy male rats. Therefore, inhibition of TXA_2_-mediated events could be considered a potential strategy to protect the cardiovascular system. Future investigations will be necessary to determine whether or not different biomarkers, in addition to TXA_2_, are modified during different periods of ADT.

## Data Availability Statement

The original contributions generated for the study are included in the article, further inquiries can be directed to the corresponding authors.

## Ethics Statement

The studies involving human participants were reviewed and approved by Clinical Research Ethics Committee of La Paz University Hospital (Ref. HULP: PI-1204). The patients/participants provided their written informed consent to participate in this study. The animal study was reviewed and approved by Research Ethics Committee of Universidad Autónoma de Madrid according to directives 609/86 CEE and R.D. 233/88 of the Ministerio de Agricultura, Pesca y Alimentación of Spain (PROEX 202/16).

## Author Contributions

MF and FdB contributed to the intellectual design of the study. MF, FdB, and AB-S supervised the work. MÁ-M, AE, PC, MK-M, and JG contributed to the development of the research in different proportions. MF wrote the article. All authors contributed to revising the manuscript and approved the submitted version.

## Conflict of Interest

The authors declare that the research was conducted in the absence of any commercial or financial relationships that could be construed as a potential conflict of interest.
